# Assessment of the prevalence of pain, adequacy of pain management and influencing factors in patients undergoing radiotherapy

**DOI:** 10.3332/ecancer.2022.1483

**Published:** 2022-12-02

**Authors:** Kurl E Jamora, Michelle Regina L Castillo, Miriam Joy C Calaguas

**Affiliations:** 1 Division of Radiation Oncology, Department of Radiology, University of the Philippines-Philippine General Hospital, Taft Avenue, Ermita, Manila 1000, Philippines

**Keywords:** pain, pain management, radiation

## Abstract

Pain is prevalent among patients with cancer who are being treated with radiotherapy. However, the prevalence of pain varies across regions, and pain management is affected by several factors. This cross-sectional study aims to determine the prevalence of pain, assess the adequacy of pain management and identify factors affecting pain in patients undergoing radiotherapy. A total of 94 patients were included in the study. The prevalence of pain was determined through the Brief Pain Inventory tool, while the adequacy of pain management was assessed through the Pain Management Index. Demographic, clinical and treatment-related factors were obtained and analysed for association with the presence of pain and the adequacy of pain management. Of the 94 patients, 59 (62.8%) experienced pain while 35 (47.2%) did not. The mean pain intensity score of patients was 3.6 (standard deviation: 2.3). Most patients (67.8%) experienced mild pain with low pain interference (67.8%) on daily functions. Of the 59 patients who experienced pain, 34 (57.6%) had inadequate pain relief while 25 (42.2%) had adequate pain control. Being admitted at the hospital during radiotherapy was significantly associated with adequate pain relief. Use of analgesic was also significantly associated with pain management, with a higher rate of weak and strong opioid use in those with adequately treated pain. In this single-institution study, the prevalence of pain was high. Pain management was inadequate in more than half of the patients experiencing pain. A disparity in the prescription of analgesics, particularly opioids, was observed. Patients with inadequate pain management were less likely to receive opioids, which likely reflects the presence of several barriers that limit its access to patients.

## Background

Pain is the most recurrent and debilitating symptom of advanced and metastatic malignancies [1]. In fact, up to 70% of cancer patients will experience pain [2], and 17%–70% of them report this as severe [3].

In 1986 (revised in 1996), the World Health Organization (WHO) developed the WHO analgesic ladder for pain control in order to decrease the prevalence of inadequate analgesia [4]. The guidelines include suggestions regarding the type of analgesics that can be prescribed for pain of varying intensities – whether mild, moderate or severe. For mild pain, a non-steroidal anti-inflammatory drug (NSAID) or acetaminophen may be given. Weak opioids such as codeine should be prescribed for moderate pain, while strong opioids (morphine, hydromorphone, oxycodone, fentanyl) should be given for severe pain.

Assessing the inadequacy of pain relief is important as it is a predictor of functional impairment [5]. The Pain Management Index (PMI) is a well-validated technique used to assess the adequacy of pain management for cancer patients [6]. It is a conservative measure based on the Agency for Health Care Policy and Research and WHO guidelines [7]. In the Philippines, numerous challenges to optimum cancer pain management have been described; some of which include decreased prevalence of opioid use, regulatory barriers, limited funding and unavailability of local pain management guidelines [8].

A large proportion of patients referred to our service for radiotherapy whether in the adjuvant, definitive or palliative setting experience pain. Apart from the pain brought by the disease itself, radiotherapy also contributes to the experience through its various acute and late toxicities. Long waiting time, treatment manipulation and daily treatment are some of the treatment-related factors that may cause further pain to patients. Identifying factors influencing pain will help us in implementing strategies to alleviate the pain of our patients and enhance their quality of life (QOL).

The objectives of this study are to determine the prevalence of pain, to assess the adequacy of pain management and to identify factors affecting pain in patients with cancer referred to our service for radiotherapy.

## Methods

This was a cross-sectional, non-interventional, prospective study conducted in a period of 2 days separated by 3 months to ensure no repeated participation from the patients. The study was conducted on 4 April and 19 July 2022 at the Division of Radiation Oncology, University of the Philippines-Philippine General Hospital. All patients treated on 4 April and 19 July who were eligible for the study were included. Patients were eligible for inclusion if they were at least 18 years old, were undergoing radiotherapy regardless of type or stage of disease and were willing to participate in the interview. Patients were excluded if they had any unstable psychiatric or mental condition. After obtaining written informed consent from the patients, demographic, clinical and treatment-related information were gathered on a case record sheet. A total of 94 patients were included. On 4 April 2022, a total of 48 patients were treated in the institution, of which 39 were included. Four paediatric patients were excluded, while five patients did not consent to the study. On 19 July 2022, a total of 72 patients were treated, of which 55 were included. Five paediatric patients were excluded, while seven patients declined to participate and five patients who underwent radiotherapy were missed for inclusion. This yielded a response rate of 77%.

Patients were then interviewed using the Brief Pain Inventory (BPI) tool. The BPI tool assesses the severity of pain (BPI pain score) and its impact on the daily functions of patients (BPI pain interference). Rating of pain was from 0 (no pain) to 10 (pain as bad as you can imagine). For the interpretation of scores, the BPI pain score categorisation was 0 for absence of pain, 1 to 4 for mild pain, 5 to 6 for moderate pain and 7 to 10 for severe pain [9]. BPI pain interference was scored as the mean of seven interference items, including general activity, mood, walking ability, normal work, relations with others, sleep and enjoyment of life. Rating of degree of interference was from 0 (does not interfere) to 10 (completely interferes). For the interpretation of scores, the BPI interference score was 1–4 for low and 5–10 for high.

Data on the PMI score was then obtained. The PMI score is a simple index that usually indicates how well the reported pain is managed by the prescribed analgesics [10]. Calculation of the PMI score involves two variables: pain score and analgesic score, both of which can be derived from the BPI questionnaire. The ‘worst’ pain score from the BPI was categorised as mild (1–4), moderate (5–6) and severe (7–10). In calculating the PMI score, a pain score of 0 was defined as an absence of pain, 1 as mild pain, 2 as moderate pain and 3 as severe pain.

A patient’s analgesic score was calculated according to the type of analgesic the patient is currently taking. A score of 0 was assigned to no analgesic medication, 1 to non-opioids such as NSAIDs or paracetamol, 2 to weak opioids (tramadol or codeine) and 3 to strong opioids (fentanyl, morphine, oxycodone or methadone). To calculate the PMI score, the pain score was subtracted from the analgesic score. Depending on the values ranging from −3 to 3, pain management was determined to be adequate or inadequate. A negative PMI score corresponded to inadequate pain management while a score of 0 or greater corresponded to adequate pain management.

Demographic, clinical and treatment-related characteristics were summarised through descriptive statistics. Frequency, proportion, mean and standard deviation (SD) were computed wherever appropriate. Data were not assessed to determine distribution. Regression analyses was performed to assess the association, and their statistical significance, of obtained variables with presence or absence of pain and in cases where pain is present, whether pain is adequately or inadequately managed. Statistical significance was set at *p* < 0.05.

The protocol was approved by the research ethics board of the University of the Philippines-Manila (UPMREB 2021-0653-01).

## Results

### Demographic, clinical and treatment-related characteristics

A total of 94 patients were enrolled in the study. Table 1 shows their demographic and clinical characteristics in relation to the presence or absence of pain. Patients were grouped according to age, gender, marital status, educational and income level, and treated location in the body. Of the 94 patients, 59 (62.8%) experienced pain while 35 (37.2%) did not. The mean age of all patients was 50 ± 13.8 years. Fifty-five percent of patients were >50 years of age. Majority were females (56.4%) and were married (57.4%). Most were able to attain some degree of college education (47.9%) and had a monthly income of <P9,250 (56.4%). Majority were treated in the head and neck (31.9%), followed by the pelvis (26.6%) and the breast or chest wall region (14.9%). None of the mentioned variables were significantly different between the two groups.

Table 2 shows the patients’ treatment and radiotherapy-related characteristics, which included treatment intent, mean dose, presence of concurrent chemotherapy, sequence of treatment, objective and subjective waiting time, treatment period, type of transportation and distance of residence from the facility. Majority of the patients were treated with a radical intent (85.1%) and received radiotherapy alone (58.5%). In terms of the waiting time for radiotherapy treatment, most objectively waited for <2 hours (53.2%) and the majority subjectively considered this as short (50%). At the time of survey, most were in the middle of treatment (24.5%) and a good number were taking public transportation (78.7%) to reach the radiotherapy facility (78.7%). Similarly, none of the explored treatment variables were significantly associated with the presence or absence of pain.

### Pain experience

**Figure 1.**
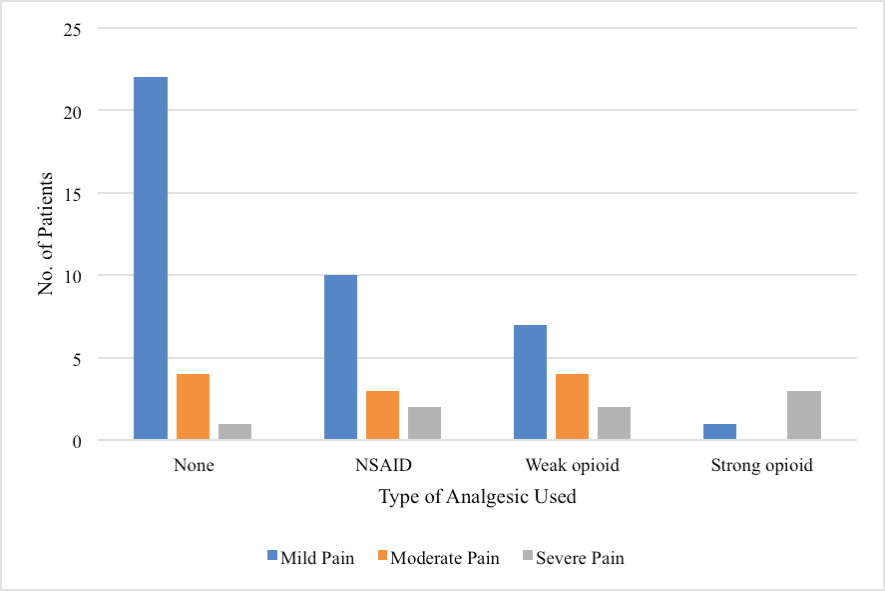
Analgesic use pattern in 59 patients experiencing pain. NSAID, Nonsteroidal anti-inflammatory drug.

The mean pain intensity score of the patients was 3.6 (SD: 2.3). BPI scores were used to evaluate the severity of pain and the impact of pain on the daily functions of patients. According to the BPI pain score, 40 patients (67.8%) experienced mild pain, 11 patients (18.6%) had moderate pain and 8 patients (13.6%) had severe pain. The BPI pain interference score revealed that 40 patients (67.8%) suffered low pain interference on their daily activities while 19 (32.2%) experienced high pain interference (Table 3). Table 4 and Figure 1 characterise the pattern of analgesic use of 59 patients in the study. Of the 27 patients with pain and no analgesic, 22 had mild pain, 4 had moderate pain and 1 had severe pain. Likewise, most of the patients with mild pain were not taking any form of analgesic. A total of four patients were on strong opioids, three of which were still experiencing severe pain.

### Adequacy of pain relief

Of the 59 patients (62.8%) who experienced pain, 34 (57.6%) had inadequate pain relief while 25 (42.2%) had adequate pain control. The association between the adequacy of pain management with patient and treatment characteristics is listed in Tables 5 and 6, respectively. Most of the clinical and treatment-related characteristics evaluated had no clear association with the adequacy of pain management except for transportation. Being admitted at the hospital during radiotherapy was significantly associated with pain relief. There was no difference in the severity of pain experienced by those who had adequate or inadequate pain relief. Whereas all patients with adequately treated pain were taking an analgesic, 79.4% of patients with undertreated pain were not. Moreover, increased rates of prescription with weak (44% versus 5.9%) and strong (16% versus 0%) opioids were observed in those with adequate pain relief.

## Discussion

In this single institution study, we noted a high prevalence (62.8%) of pain in 94 oncologic patients undergoing radiotherapy. Among these, the majority experienced mild pain (67.8%) with low pain interference (67.8%) on day-to-day personal life. Strikingly, more than half (57.6%) of patients who experienced pain were inadequately managed. Most of the patients with mild pain were not on any analgesic, and strong opioids were prescribed to only a few number of patients.

The prevalence of pain in our study population is consistent with that of published data indicating a 59% prevalence of pain in patients undergoing oncologic treatment and a 64% prevalence in patients characterised as advanced or metastatic [11]. In contrast, the rates of undertreated pain in our study appear to be higher than reported in literature. A systematic review published in 2008 revealed a 43.4% prevalence of inadequately treated cancer-related pain as assessed by the PMI tool [12]. Furthermore, in an update released in 2014, there was a noticeable decrease in undertreatment of approximately 25% (from 43.4% to 31.8%) [13]. Nevertheless, when considering geographic and economic aspects, our results appear to parallel that of Asian statistics. In the previously cited updated systematic review, the Asia group reported the highest rates of negative PMI scores (corresponding to inadequate analgesia) from 1997 to 2007 (59.1%). In 1989–1990, a survey in the Philippines showed that three-fourths of cancer patients suffered pain, and among these, 60% were unrelieved [14].

The high prevalence of pain and inadequate pain management in our cohort of patients would likely have negative implications in their outcomes. Inadequately treated pain can decrease compliance of patients to radiotherapy which may result to treatment interruptions or deficiencies. Treatment interruptions have been shown to adversely impact outcomes in a number of disease sites [15–17]. More importantly, inadequately managed pain would possibly worsen the QOL of patients [18]. It could affect their mobility, disrupt their sleep and decrease their productivity. This could augment existing negative perceptions toward pain and affect health-seeking attitudes. Although measuring the compliance of patients and their QOL are out of the scope of this study’s aims, these outcomes were likely influenced as a result of pain and are worth exploring in the future.

The high prevalence of pain and inadequate pain management in our cohort of patients may have some cultural implications. In an article on Filipino attitudes toward pain medication, Galanti [19] stated that Filipinos are characteristically stoic in painful situations. Some have a high tolerance to pain and are apprehensive of becoming addicted to narcotics. This cultural perception towards pain and pain medications potentially explains how patients ‘endure’ pain.

In our study, the use of analgesic was significantly associated with adequate pain relief, with a higher rate of weak and strong opioid use in those with adequately treated pain. As the use of opioids occupy two steps of the WHO ladder in pain management, its use or non-use could significantly impact the adequacy of analgesia of our cancer patients. Various local studies have mainly shown that a barrier to an optimal pain control in the Philippines is the infrequent use of these medications. In our study, only four patients were prescribed with strong opioids despite almost a third of patients experiencing moderate to severe pain. In the Philippines, several regularity barriers to accessibility of strong opioids include burdensome procedures relating to prescription, physician prescriber restrictions, requirement for special prescription forms and excessive bureaucratic regulation policies [20, 21]. Although our country has all six formulations of opioid available in the national formulary, these were available to patients only half the time (as opposed to 70% of the time for other Southeast Asian countries). While other countries have increased opioid usage over the years, ours has remained largely the same [8]. In most countries, pain medications are also provided for free or at a reduced cost (<25%). However, in the Philippines, patients shoulder the full cost of these medicines [22].

Another aim of this study was to assess factors related to radiation oncology practice that might influence pain. This has been previously investigated in a radiotherapy unit in France. Their results showed that objective length of waiting time for a session, transportation and mobilisation for a session positioning significantly aggravated the pain of a considerable number of patients [23]. In our study, a factor found to influence the adequacy of pain management is transportation. Transportation has been shown to be a barrier to adequate pain relief in some studies [24, 25]. Transportation barriers were more likely experienced by patients with uncontrolled pain. In our study, the distance from the place of residence to the radiotherapy facility as well as the mode of transportation (whether public or private) did not correlate with the adequacy of pain management. However, it appeared that patients who were admitted were more likely to be adequately treated. It is probable that these patients were assessed more regularly by their attending physicians in the wards and that they had more access to pain medications while admitted.

It should be noted however that despite the high prevalence of pain and inadequate pain management in our cohort, most of these patients experienced mild pain with low pain interference. Forty (40) patients experienced mild pain and 22 of them were not taking any analgesics.

These patients comprise 65% (22 out of 34) of patients with inadequately treated pain. In addition, 28 of patients with inadequately treated pain had a PMI score of −1, whereas 6 patients had a PMI score of −2. The PMI has its own criticisms. A prospective observational study has shown that PMI scores of −1 do not always indicate inadequate pain management. The study revealed that patients with adequate pain management (particularly those with PMI score of 0) have higher pain interference than those with a PMI score of −1 [26]. Furthermore, patients with mild pain may be managed with other non-NSAID or non-paracetamol analgesics such as pregabalin or steroids. Any patients treated with a strong opioid will also be considered as adequately treated despite presence of severe pain as it does not take into account the dosage of medications [27]. Nevertheless, the PMI tool has been frequently reported as giving extensive proof of its validity.

Further studies to characterise the origin of pain of patients (i.e. whether from the disease, acute toxicity of treatment or manipulation-related), the prescription practice of physicians and the attitudes of patients toward pain and pain medications are recommended. Assessing these variables will aid in formulating strategies to address the high prevalence of pain. A high proportion of pain due to radiation-related toxicities could motivate more regular screening of side effects experienced by patients during treatment as well as more access to healthcare personnel for pain-related concerns. On the other hand, a high proportion of pain due to treatment manipulation can alert the radiotherapy personnel on the implementation of practices that ensure comfort of patients. It is also important to evaluate the knowledge and practice of physicians and oncologists in pain and pain management, including their possession of a special licence for narcotics prescription and their pattern of prescribing analgesics to their patients. As a rigorous process of narcotics license application is one of the identified barriers to optimal cancer pain management, determining the proportion of physicians in the institution who have this license is vital. Collaboration with government agencies to setup satellite hubs in the hospital to guide licence application could be organised. Furthermore, refresher lectures on pain management could be facilitated. Lastly, determining the attitudes of patients towards pain and pain management is vital to addressing the issue. A regular forum or focused group discussion could be assembled to tackle misconceptions on pain and pain medications and to encourage positive health-seeking behaviour. It is also equally important to understand the social factors that play into the attitudes and practices of patients towards pain including financial implications and social support.

## Conclusion

In this single institution study, the prevalence of pain was high. Pain management was inadequate in more than half of the patients experiencing pain. A disparity in prescription of analgesics, particularly opioids, was observed among those with adequately treated and inadequately treated pain.

As undertreatment of cancer-related pain remains a common problem worldwide, it is important to determine factors that hamper provision of optimal care even at an institutional level. This study characterises the pain and the pattern of analgesic use in a small cohort of patients. It highlights the socioeconomic factors, cultural influences and decreased prevalence of opioid use as potential barriers to pain management. Despite the availability of opioids in our national formulary, several barriers exist that limit its access to patients including regulatory restrictions and prohibitive costs. To address these, a collective effort from all stakeholders is imperative. Additionally, it may be beneficial to evaluate the knowledge and practice of physicians regarding pain management and to investigate further the perception and attitudes of patients towards pain medications.

## Conflicts of interest

The authors declare that they have no conflicts of interest.

## Funding

The authors received no financial support for the research, authorship and/or publication of this article.

## Figures and Tables

**Table 1.** Demographic and clinical characteristics of patients with and without pain (*n* = 94).

	Pain	No pain	Total patient population	*p*-value
	*n* = 59	*n* = 35		
	*n* (%)	*n* (%)	*n* (%)	
Mean age (years)	50.27 ± 14.06	50.31 ± 13.46		0.9892
Age group				0.482
≤50 years	28 (47.5)	14 (40)	42 (44.7)	
>50 years	31 (52.5)	21 (60)	52 (55.3)	
Gender				0.586
Male	27 (45.8)	14 (40)	41 (43.6)	
Female	32 (54.2)	21 (60)	53 (56.4)	
Marital status				0.658
Single	15 (25.4)	12 (34.3)	27 (28.7)	
Married	34 (57.6)	20 (57.1)	54 (57.4)	
Widow/er	6 (10.2)	1 (2.9)	7 (7.4)	
Separated	3 (5.1)	1 (2.9)	4 (4.3)	
Live-in	1 (1.7)	1 (2.9)	2 (2.1)	
Educational level				0.191
Elementary	9 (15.3)	1 (2.9)	10 (10.6)	
High school	21 (35.6)	15 (0)	36 (38.3)	
College	28 (47.5)	17 (48.6)	45 (47.9)	
Post-baccalaureate	1 (1.7)	2 (5.7)	3 (3.2)	
Income level				0.276
Less than P9,250	37 (62.7)	16 (45.7)	53 (56.4)	
Between P9,250 and 19,040	11 (18.6)	13 (37.1)	24 (25.5)	
Between P19,040 and 38,080	6 (10.2)	3 (8.6)	9 (9.6)	
Between P38,080 and 66,640	3 (5.1)	2 (5.7)	5 (5.3)	
Between P66,640 and 114,240	1 (1.7)	0 (0)	1 (1.1)	
Between P114,240 and 190,400	0 (0)	1 (2.9)	1 (1.1)	
At least P190,400	1 (1.7)	0 (0)	1 (1.1)	
Treated location in the body				0.373
Head and neck	24 (40.7)	6 (17.1)	30 (31.9)	
Brain (primary or metastases)	6 (10.2)	7 (20)	13 (13.8)	
Breast/chest wall/thorax	8 (13.6)	6 (17.1)	14 (14.9)	
Pelvis	15 (25.4)	10 (28.6)	25 (26.6)	
Bone metastases	2 (3.4)	1 (2.9)	3 (3.2)	
Abdomen	2 (3.4)	1 (2.9)	3 (3.2)	
Oesophagus	1 (1.7)	0 (0)	1 (1.1)	
Extremity	1 (1.7)	0 (0)	1 (1.1)	

**Table 2.** Treatment and radiotherapy-related characteristics of patients with and without pain (*n* = 94).

	Pain	No pain	Total populatio*n*	*p*-value
	*n* = 59	*n* = 35		
	*n* (%)	*n* (%)	*n* (%)	
Treatment intent				0.467
Radical	49 (83.1)	31 (88.6)	80 (85.1)	
Palliative	10 (16.9)	4 (11.4)	14 (14.9)	
Mean dose (Gy)	28.45 ± 19.27	21.94 ± 18.41		0.111
Radiotherapy alone				0.740
Postoperative	12 (20.3)	9 (25.7)	21 (22.3)	
After chemotherapy	5 (8.5)	6 (17.1)	11 (11.7)	
Preoperative	0 (0)	1 (2.9)	1 (1.1)	
Before chemotherapy	2 (3.4)	1 (2.9)	3 (3.2)	
Only	12 (20.3)	7 (20)	19 (20.2)	
Concurrent chemoradiation				0.231
Postoperative	5 (8.5)	4 (11.4)	9 (9.6)	
After chemotherapy	6 (10.2)	1 (2.9)	7 (7.4)	
Preoperative	0 (0)	1 (2.9)	1 (1.1)	
Before chemotherapy	0 (0)	0 (0)	0 (0)	
Only	17 (28.8)	5 (14.3)	22 (23.4)	
Hour of treatment				0.183
6:00–9:00	11 (18.6)	8 (22.9)	19 (20.2)	
9:00–12:00	11 (18.6)	8 (22.9)	19 (20.2)	
12:00–15:00	9 (15.3)	8 (22.9)	17 (18.1)	
15:00–18:00	18 (30.5)	3 (8.6)	21 (22.3)	
18:00 and beyond	10 (16.9)	8 (22.9)	18 (19.1)	
Objective waiting time				0.911
<2 hours	31 (52.5)	19 (54.3)	50 (53.2)	
≥2 hours	28 (47.5)	16 (45.7)	44 (46.8)	
Subjective waiting time				>0.999
Very long	5 (8.5)	2 (5.7)	7 (7.4)	
Long	24 (40.7)	14 (40)	38 (40.4)	
Short	29 (49.2)	18 (51.4)	47 (50)	
Very short	1 (1.7)	1 (2.9)	2 (2.1)	
Treatment period in radiotherapy				0.293
Beginning	8 (13.6)	11 (31.4)	19 (20.2)	
First quarter	13 (22)	6 (17.1)	19 (20.2)	
Half	15 (25.4)	8 (22.9)	23 (24.5)	
Third quarter	13 (22)	7 (20)	20 (21.3)	
End	10 (16.9)	3 (8.6)	13 (13.8)	
Transportation				0.805
Private	11 (18.6)	7 (20)	18 (19.1)	
Public	46 (78)	28 (80)	74 (78.7)	
Admitted	2 (3.4)	0 (0)	2 (2.1)	
Place of residence (mean kilometre**)**	16.61 ± 14.48	16.46 ± 9.82		0.957

**Table 3.** Scoring the BPI as an outcome measure.

BPI pain score	No. (%)	Average score
Mild (1–4)	40 (67.8)	2.3
Moderate (5–6)	11 (18.6)	5.2
Severe (7–10)	8 (13.6)	8
BPI interference score		
Low (1–4)	40 (67.8)	2.2
High (5–10)	19 (32.2)	7.3

**Table 4.** Details of analgesic treatment in relation to severity of pain.

Analgesic type	Pain severity			Total
	Mild	Moderate	Severe	
No analgesic	22	4	1	27 (45.8)
NSAID	2			2 (3.4)
Paracetamol	8	3	1	12 (20.3)
Paracetamol and NSAID			1	1 (1.7)
Tramadol	6	2	1	9 (15.3)
Tramadol and paracetamol/NSAID	1	2	1	4 (6.8)
Morphine			3	3 (5.1)
Oxycodone and fentanyl patch	1			1 (1.7)
Total	40 (67.8)	11 (18.6)	8 (13.6)	59

**Table 5.** Demographic and clinical characteristics of patients with adequate and inadequate pain relief (*n* = 59).

	Pain relief		*p*-value
	Adequate (*n* = 25)	Inadequate (*n* = 34)	
	*n* (%)	*n* (%)	
Mean age (years)	49 ± 15.09	51.21 ± 13.40	0.555
Age group			0.943
≤50 years	12 (48)	16 (47.1)	
>50 years	13 (52)	18 (52.9)	
Gender			0.176
Male	14 (56)	13 (38.2)	
Female	11 (44)	21 (61.8)	
Marital status			0.082
Single	10 (40)	5 (14.7)	
Married	12 (48)	22 (64.7)	
Widow/er	1 (4)	5 (14.7)	
Separated	1 (4)	2 (5.9)	
Live-in	1 (4)	0 (0)	
Educational			0.050
Elementary graduate	8 (32)	1 (2.9)	
High school graduate	9 (36)	12 (35.3)	
College graduate	8 (32)	21 (61.8)	
Post-baccalaureate	0 (0)	0 (0)	
Income level			0.751
Less than P9,250	18 (72)	19 (55.9)	
Between P9,250 and 19,040	3 (12)	8 (23.5)	
Between P19,040 and 38,080	3 (12)	3 (8.8)	
Between P38,080 and 66,640	1 (4)	2 (5.9)	
Between P66,640 and 114,240	0 (0)	1 (2.9)	
Between P114,240 and 190,400	0 (0)	0 (0)	
At least P190,400	0 (0)	1 (2.9)	
Treated location in the body			0.685
Head and neck	11 (44)	13 (38.2)	
Brain (primary or metastases)	4 (16)	2 (5.9)	
Breast/chest wall/thorax	3 (12)	5 (14.7)	
Pelvis	7 (28)	8 (23.5)	
Bone metastases	0 (0)	2 (5.9)	
Abdomen	0 (0)	2 (5.9)	
Oesophagus	0 (0)	1 (2.9)	
Extremity	0 (0)	1 (2.9)	

**Table 6.** Treatment and radiotherapy-related characteristics of patients with adequate and inadequate pain relief (*n* = 59).

	Pain relief		*p*-value
	Adequate (n = 25)	Inadequate (n = 34)	
	*n* (%)	*n* (%)	
Treatment intent			>0.999
Radical	21 (84)	28 (82.4)	
Palliative	4 (16)	6 (17.6)	
Mean dose (Gy)	24.37 ± 16.90	31.61 ± 20.19	0.151
Radiotherapy alone			0.475
Postoperative	3 (12)	8 (23.5)	
After chemotherapy	1 (4)	4 (11.8)	
Preoperative	1 (4)	0 (0)	
Before chemotherapy	1 (4)	1 (2.9)	
Only	6 (24)	6 (17.6)	
Concurrent chemoradiation			0.860
Postoperative	1 (4)	1 (2.9)	
After chemotherapy	2 (8)	4 (11.8)	
Preoperative	1 (4)	2 (5.9)	
Before chemotherapy	0 (0)	0 (0)	
Only	9 (36)	8 (23.5)	
Hour of treatment			0.471
6:00–9:00	4 (16)	7 (20.6)	
9:00–12:00	3 (12)	8 (23.5)	
12:00–15:00	5 (20)	4 (11.8)	
15:00–18:00	10 (40)	8 (23.5)	
18:00 and beyond	3 (12)	7 (20.6)	
Objective waiting time (mean, h:m)			0.943
<2 hours	13 (52)	18 (52.9)	
≥2 hours	12 (48)	16 (47.1)	
Subjective waiting time			0.409
Very long	1 (4)	0 (0)	
Long	9 (36)	15 (44.1)	
Short	14 (56)	15 (44.1)	
Very short	1 (4)	4 (11.8)	
Treatment period in radiotherapy			0.304
Beginning	3 (12)	5 (14.7)	
First quarter	9 (36)	4 (11.8)	
Half	5 (20)	10 (29.4)	
Third quarter	5 (20)	8 (23.5)	
End	3 (12)	7 (20.6)	
Transportation			0.022
Private	1 (4)	9 (26.5)	
Public	21 (84)	25 (73.5)	
Admitted	2 (8)	0 (0)	
Place of residence (mean kilometre)	16.98 ± 14.84	16.35 ± 14.44	0.871
Pain severity			0.924
Mild	18 (72)	22 (64.7)	
Moderate	4 (16)	7 (20.6)	
Severe	3 (12)	5 (14.7)	
Analgesic			<0.001
None	0 (0)	27 (79.4)	
NSAID/paracetamol	10 (40)	5 (14.7)	
Weak opioid	11 (44)	2 (5.9)	
Strong opioid	4 (16)	0 (0)	
